# Increased Circulating miR-21 Levels Are Associated with Kidney Fibrosis

**DOI:** 10.1371/journal.pone.0058014

**Published:** 2013-02-28

**Authors:** François Glowacki, Grégoire Savary, Viviane Gnemmi, David Buob, Cynthia Van der Hauwaert, Jean-Marc Lo-Guidice, Sébastien Bouyé, Marc Hazzan, Nicolas Pottier, Michaël Perrais, Sébastien Aubert, Christelle Cauffiez

**Affiliations:** 1 EA4483, Département de Biochimie et Biologie Moléculaire, Faculté de Médecine H Warembourg, Pôle Recherche, Lille, France; 2 Service de Néphrologie, Hôpital Huriez, CHRU, Lille, France; 3 Institut National de la Santé et de la Recherche Médicale, U837, Jean-Pierre Aubert Research Center, Equipe 5 «Mucines, Différenciation et Cancérogenèse Épithéliales», Lille, France; 4 Institut de Pathologie, Centre de Biologie Pathologie Génétique, CHRU, Lille, France; 5 Faculté de Médecine H Warembourg, Université Lille 2, Lille, France; 6 EA2686, Faculté de Médecine H Warembourg, Pôle Recherche, Université Lille 2, Lille, France; 7 Service d'Urologie, Hôpital Huriez, CHRU, Lille, France; University of Washington, United States of America

## Abstract

MicroRNAs (miRNAs) are a class of noncoding RNA acting at a post-transcriptional level to control the expression of large sets of target mRNAs. While there is evidence that miRNAs deregulation plays a causative role in various complex disorders, their role in fibrotic kidney diseases is largely unexplored. Here, we found a strong up-regulation of miR-21 in the kidneys of mice with unilateral ureteral obstruction and also in the kidneys of patients with severe kidney fibrosis. In addition, mouse primary fibroblasts derived from fibrotic kidneys exhibited higher miR-21 expression level compared to those derived from normal kidneys. Expression of miR-21 in normal primary kidney fibroblasts was induced upon TGFβ exposure, a key growth factor involved in fibrogenesis. Finally, ectopic expression of miR-21 in primary kidney fibroblasts was sufficient to promote myofibroblast differentiation. As circulating miRNAs have been suggested as promising non-invasive biomarkers, we further assess whether circulating miR-21 levels are associated with renal fibrosis using sera from 42 renal transplant recipients, categorized according to their renal fibrosis severity, evaluated on allograft biopsies (Interstitial Fibrosis/Tubular Atrophy (IF/TA). Circulating miR-21 levels are significantly increased in patients with severe IF/TA grade (IF/TA grade 3: 3.0±1.0 *vs* lower grade of fibrosis: 1.5±1.2; p = 0.001). By contrast, circulating miR-21 levels were not correlated with other renal histological lesions. In a multivariate linear regression model including IF/TA grade and estimated GFR, independent associations were found between circulating miR-21 levels and IF/TA score (ß = 0.307, p = 0.03), and between miR-21 levels and aMDRD (ß = −0.398, p = 0.006). Altogether, these data suggest miR-21 has a key pathogenic role in kidney fibrosis and may represent a novel, predictive and reliable blood marker of kidney fibrosis.

## Introduction

Renal fibrosis, the final common end stage of a wide variety of chronic kidney diseases, is characterized by the excessive and persistent accumulation of extracellular matrix (ECM) proteins responsible for the progressive destruction of kidney parenchyma [1]–[2]. In particular, kidney transplantation may be considered as an *in vivo* model of accelerated interstitial kidney fibrosis [3]. Indeed, after renal transplantation, several processes involving both alloantigen-dependent and independent factors induce chronic allograft dysfunction and consequently a renal change characterized notably by interstitial fibrosis (IF), tubular atrophy (TA) and glomerular sclerosis [4].

Kidney fibrosis is a complex disorder whose underlying molecular mechanisms remain largely unknown. Irrespective of the initial cause (infectious, autoimmune, toxic, obstructive…), the current paradigm suggests that aberrant wound healing of the kidney tissue following the sustained injury is the key driving process of the fibrotic response. Indeed, after the initial insult, the affected kidney tissue undergoes a cascade of molecular events to restore tissue integrity. These processes include in particular the activation of kidney resident cells followed by the release of proinflammatory cytokines along with the subsequent infiltration of inflammatory monocytes/macrophages and T cells to the injured sites. Depending on the etiology of renal injury and if the injurious condition is sustained, glomerular or interstitial infiltrated inflammatory cells become activated, and produce detrimental molecules such as pro-fibrotic and inflammatory cytokines (especially TGFβ) or reactive oxygen species [5]–[7]. Finally, mesangial cells, fibroblasts, and tubular epithelial cells are then stimulated and undergo phenotypic activation or transition and produce a large amount of ECM components. This continuous excessive deposition of ECM proteins results in fibrous scars and distorts the fine architecture of kidney tissue, ultimately leading to the loss of kidney function [8]–[9].

MiRNAs, a set of approximately 22 nucleotide noncoding RNAs, are a major class of gene expression regulators with essential functions in numerous biological processes including development, differentiation, stress response, apoptosis and proliferation [10]. Since the first miRNA was identified in *Caenorhabditis elegans* as an important factor for timing of larval development [11]–[12], about 1500 miRNAs have now been identified and/or characterized in the human genome (miRbase version 18, see http://www.mirbase.org/). To date, numerous studies have identified specific miRNA expression patterns related to the initiation and progression of various diseases including cancer as well as inflammatory, infectious and autoimmune diseases [13]–[14]. Nevertheless, the precise role of miRNA in kidney fibrosis remains largely unknown [15]–[16].

Increasing evidence also suggests that miRNA profiling is a promising approach to develop new biomarkers for diagnosis, prognosis or disease activity [17]–[18]. This line of research is best exemplified with the discovery of high correlation between circulating miRNA levels and various clinico-pathological endpoints. This, along with the remarkable stability of miRNAs in plasma and serum, underscores their powerful value as a new class of blood-based biomarkers [19]–[21] and opens new avenues for the development of non-invasive tests.

To identify miRNAs with potential roles in the development of renal fibrosis, we performed a genome-wide assessment of miRNA expression in kidneys obtained from mice with unilateral ureteral obstruction, a well-established experimental model of renal fibrosis [22]. In particular, we identified a strong up-regulation of miR-21 during the fibrogenic response to kidney injury in mice and also in renal tissue samples from patients with advanced kidney fibrosis. Therefore, since miR-21 was detected in serum, the potential of circulating miR-21 levels as a non invasive marker of kidney fibrosis was further investigated. To our knowledge, we showed for the first time that miR-21 circulating levels are significantly increased in patients affected by severe kidney fibrosis. In addition, circulating miR-21 levels were also correlated to estimated Glomerular Filtration Rate. These preliminary data suggest that circulating miR-21 expression is a potential novel and predictive marker of severe kidney fibrosis.

## Results

### miR-21 is a relevant miRNA involved in kidney fibrosis

**Figure 1:**
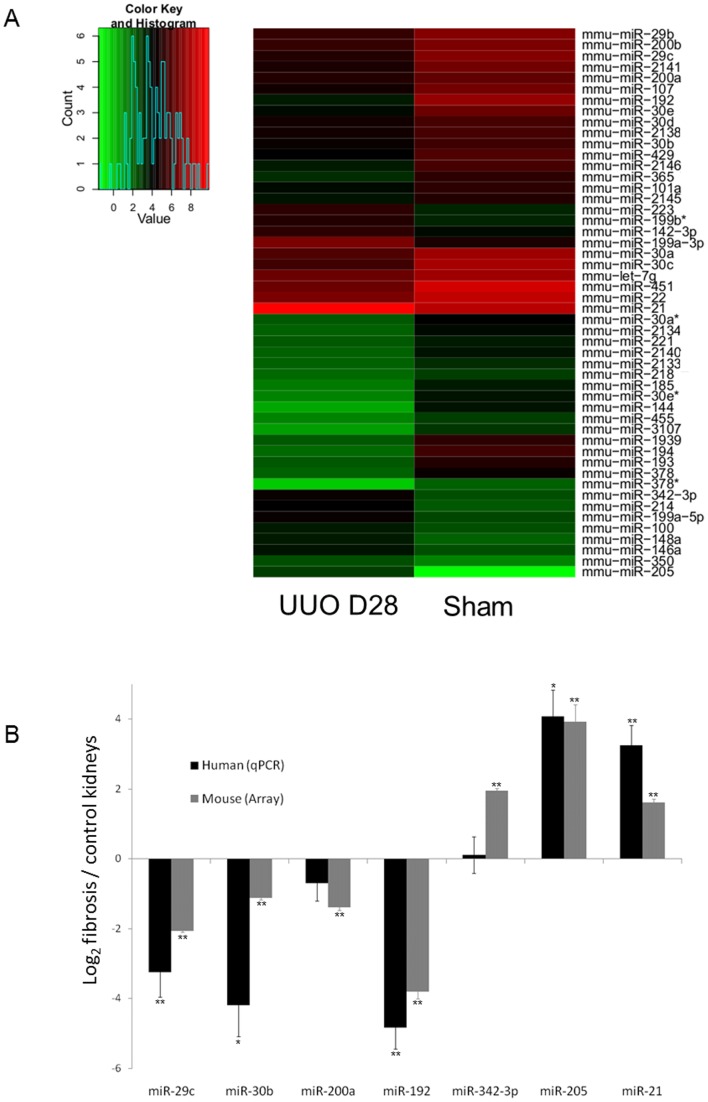
miR-21 as a relevant miRNA involved in kidney fibrosis. (A) Heat map representing the statistically significant (Bonferroni adjusted p-value <0.01) differentially expressed microRNAs in kidneys with unilateral ureteral obstruction (UUO 28 Days) compared to control kidneys (sham). n = 4 mice in each group. (B) Differential expression of miR-29c, miR-30b, miR-200a, miR-192, miR-342-3p, miR-205 and miR-21 in UUO mouse model (array) and in human fibrotic kidneys (quantitative PCR, qPCR). Results are indicated as mean of log_2_ (injured kidneys versus control kidneys). Errors are presented as SEM. Significant differences in miRNA expression between injured and control kidneys, according to array or qPCR experiments were represented as follow: *p<0.05, **p<0.01. Mice: n = 4 in each group. Human: n = 10 in each group.

In a systematic approach to identify miRNAs involved in kidney fibrosis, we compared miRNA expression profile of fibrotic kidneys from mice 28 days after Unilateral Ureteral Obstruction (UUO) to that of control kidneys using microarray. MiRNAs were considered as statistically differentially expressed between injured and control kidneys at p-value below 0.01. We identified a panel of 50 miRNAs whose expression significantly differs between normal and fibrotic kidneys (**Figure 1A** and **Table 1**) of which 37 were down-regulated and 13 were up-regulated in mouse fibrotic kidneys. Most notable, among the up-regulated miRNAs were miR-205, miR-342 and miR-21, while miR-29c, miR-192, miR-30b and miR-200a were significantly down-regulated. To assess the clinical relevance of these findings, differential expression of these seven representative miRNAs in the mouse model was further evaluated by real-time PCR in human fibrotic kidneys (**Figure 1B**). In particular, our data showed a strong up-regulation of miR-21 in the injured kidney (fold change of 3 between injured and control kidneys), a miRNA known to exert major pathogenic effects in tissue fibrosis [14], [23]–[24]. Noteworthy, in fibrotic kidneys, miR-21 exhibited the highest expression level. Then, the following experiments focused on miR-21 implication in renal fibrosis.

**Table 1:** Expression profiles of miRNAs were performed by microarray analysis and revealed 50 differentially expressed miRNAs in UUO mouse model.

	p-value	Log2 (UUO/sham)
**Down-regulated miRNA (n = 37)**		
mmu-miR-192	0.000431	−3.8048091
mmu-miR-194	0.00008	−3.48449657
mmu-miR-144	0.000891	−3.17617514
mmu-miR-1939	0.001536	−3.11770452
mmu-miR-193	0.000000	−2.78494363
mmu-miR-30e	0.000075	−2.63929985
mmu-miR-378	0.000001	−2.43765114
mmu-miR-2146	0.000024	−2.39497824
mmu-miR-30e*	0.000328	−2.3730894
mmu-miR-378*	0.000028	−2.3438244
mmu-miR-3107	0.000295	−2.23862079
mmu-miR-451	0.001343	−2.21756793
mmu-miR-30c	0.000215	−2.17793469
mmu-miR-185	0.000043	−2.13355136
mmu-miR-107	0.000001	−2.11357549
mmu-miR-365	0.000021	−2.07829141
mmu-miR-29c	0.000003	−2.05768538
mmu-miR-2134	0.000124	−1.95207121
mmu-miR-2141	0.000019	−1.82916396
mmu-miR-2140	0.000028	−1.77781433
mmu-miR-30a*	0.002073	−1.77216121
mmu-miR-29b	0.000146	−1.74096964
mmu-miR-30a	0.000137	−1.72715807
mmu-miR-429	0.00018	−1.53884554
mmu-miR-22	0.000304	−1.4779387
mmu-miR-200b	0.00192	−1.46695047
mmu-miR-200a	0.000364	−1.39479684
mmu-miR-221	0.000368	−1.37628416
mmu-miR-2145	0.000100	−1.29225451
mmu-miR-455	0.002542	−1.27395678
mmu-miR-101a	0.000056	−1.227094
mmu-miR-let-7g	0.000086	−1.22309962
mmu-miR-2138	0.000056	−1.1250415
mmu-miR-30b	0.000004	−1.12075616
mmu-miR-2133	0.000382	−1.09512979
mmu-miR-30d	0.003136	−1.08042327
mmu-miR-218	0.000175	−1.00312769
**Up-regulated miRNA (n = 13)**		
mmu-miR-205	0.000184	3.92700288
mmu-miR-199a-3p	0.000026	1.98898365
mmu-miR-342-3p	0.000027	1.95335752
mmu-miR-214	0.000015	1.94544365
mmu-miR-199a-5p	0.000000	1.88971783
mmu-miR-223	0.000019	1.76768255
mmu-miR-199b*	0.000052	1.67170497
mmu-miR-21	0.000718	1.61857791
mmu-miR-146a	0.000001	1.51438686
mmu-miR-142-3p	0.002995	1.38532666
mmu-miR-148a	0.001349	1.2935533
mmu-miR-100	0.000001	1.15643869
mmu-miR-350	0.002692	1.10017907

### Altered expression of miR-21 in fibrotic kidneys

**Figure 2:**
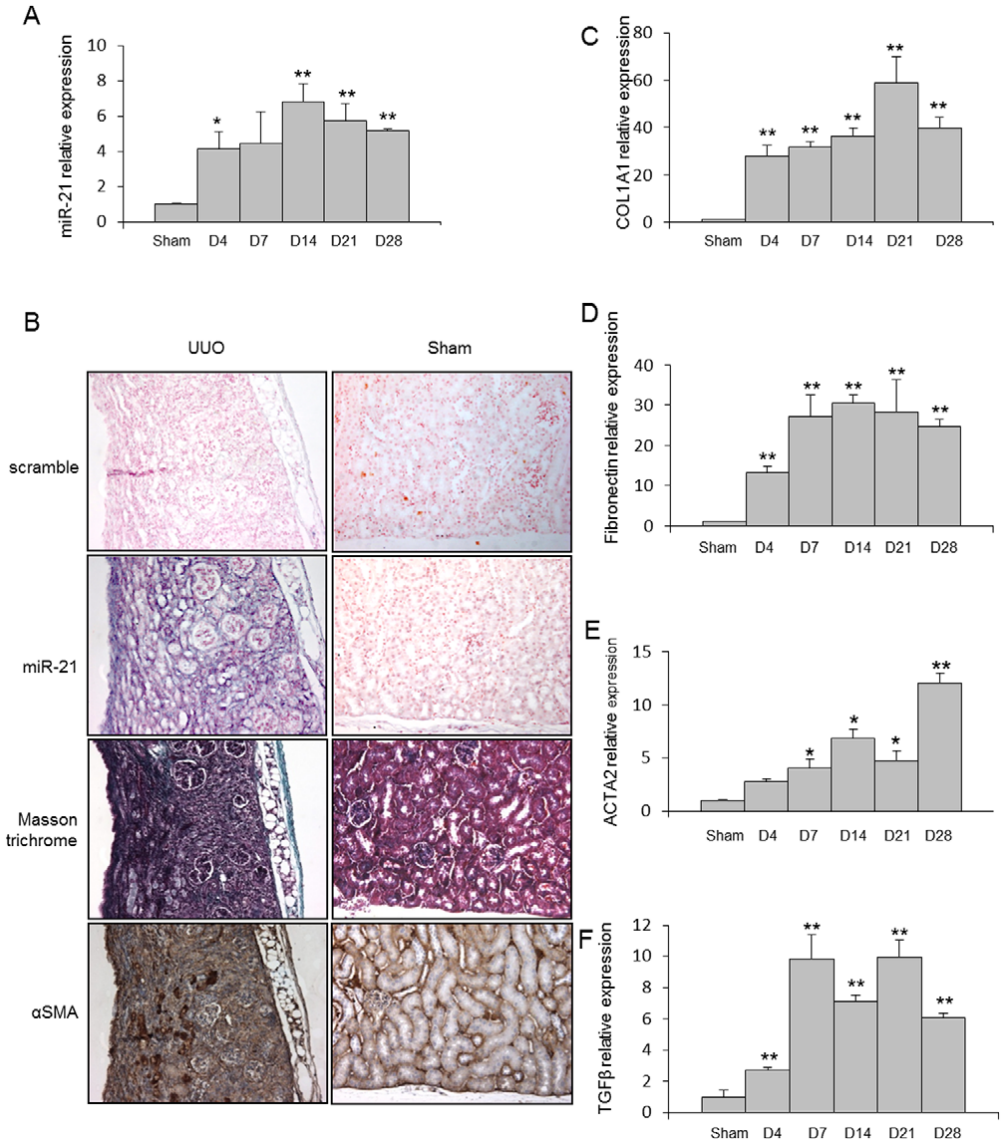
miR-21 is up-regulated in kidneys from Unilateral Ureteral Obstruction (UUO) mice. (A) miR-21 expression in kidneys from C57BL/6 mice after UUO at the indicated time points. SNO-251 was used as normalizer, n = 5 to 9 mice in each group. (B) *In situ* hybridization assay was performed to determine the stromal localization of miR-21 and its colocalization with α-SMA in kidneys of C57BL/6 mice harvested 28 days after UUO. (C) COL1A1, (D) fibronectin, (E) ACTA2 and (F) TGFβ expression in kidney from C57BL/6 mice after UUO at the indicated time points. PPIA was used as normalizer, n = 5 to 9 mice in each group. Data are expressed as mean ± SEM. * p<0.05 and ** p<0.01.

**Figure 3:**
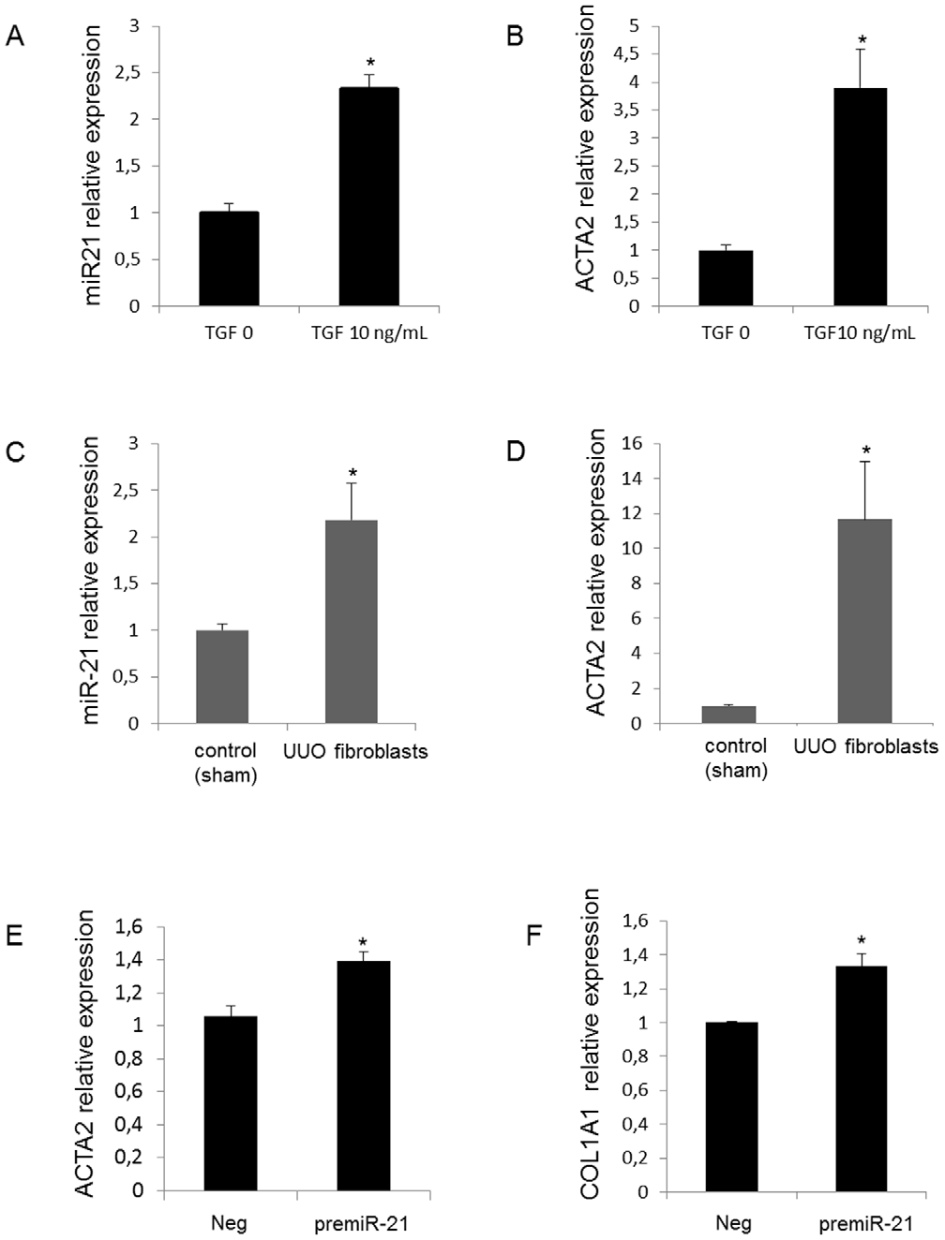
miR-21 is expressed in activated renal fibroblasts and upregulated in response to TGFβ stimulation . miR-21 and ACTA2 expression levels obtained by real time PCR in (A–B) control primary kidney fibroblasts exposed or not to TGF-β1 (10 ng/mL), and in (C–D) primary fibroblasts derived from fibrotic kidneys (UUO mice) and primary fibroblasts derived from normal kidneys (sham mice). (E–F) ACTA2 and COL1A1 expression levels obtained by real time PCR in control primary kidney fibroblasts transfected with either scrambled miRNA (neg) or premiR-21 (10 nM). Data are expressed as mean ± SEM. * p<0.05.

As depicted in **Figure 2A**, mice with UUO exhibited a time dependent increased expression of miR-21 during disease progression. *In situ* hybridization experiments performed in the injured kidneys 28 days after UUO revealed a selective expression of miR-21 in area consistent with fibrotic lesions (**Figure 2B**). In addition, miR-21 levels were correlated with the expression of two major extracellular matrix proteins (fibronectin and type I collagen, COL1A1) released by activated kidney fibroblasts, as well as TGFβ, a central pro-fibrotic growth factor, and α Smooth Muscle Actin (ACTA2), a marker of myofibroblast differentiation (**Figure 2C–F**). Furthermore, expression of miR-21 in normal primary kidney fibroblasts was significantly induced upon TGFβ exposure (**Figure 3A and 3B**). Fibroblasts derived from fibrotic kidneys (28 days UUO) exhibited higher miR-21 expression level (**Figure 3C**) and higher ACTA2 expression (**Figure 3D**) compared to control fibroblasts. Finally, we further showed that ectopic expression of miR-21 in primary kidney fibroblasts was sufficient to promote myofibroblast differentiation, as determined by an increased in both ACTA2 (**Figure 3E**) and COL1A1 (**Figure 3F**) expression levels. Altogether, these findings strongly suggest that miR-21 is likely to be involved in the pathogenic activation of kidney fibroblasts during fibrosis.

**Figure 4:**
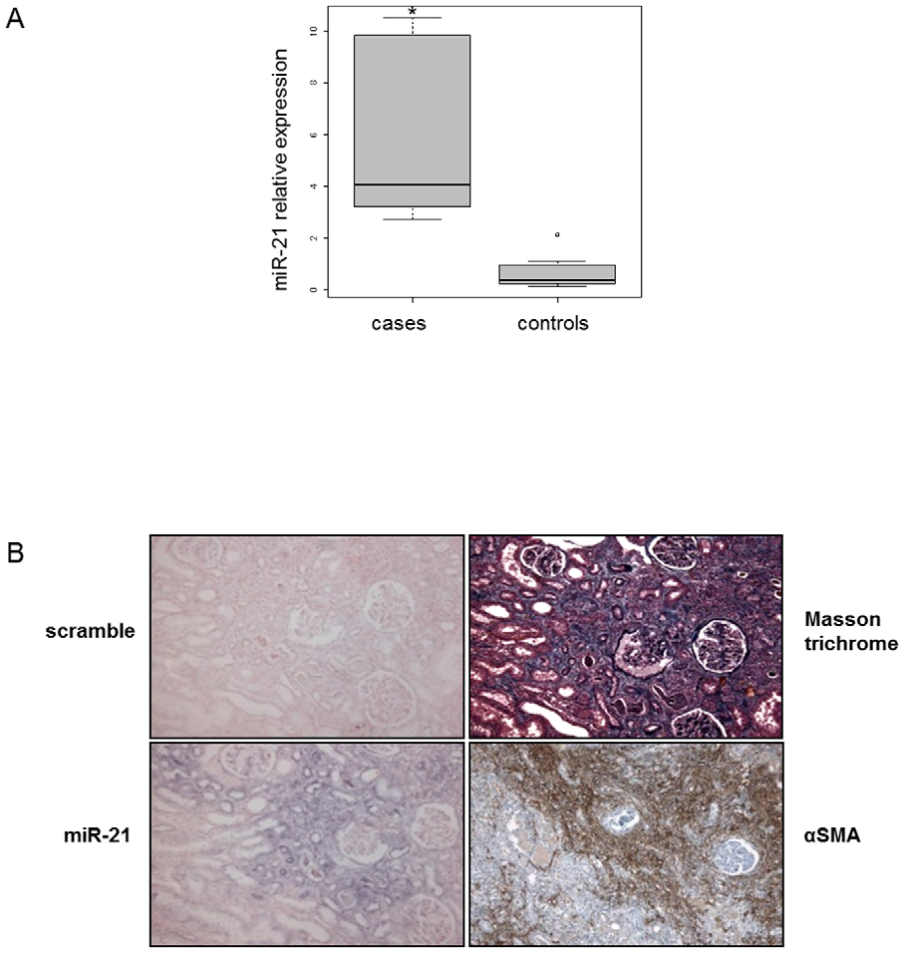
miR-21 is up-regulated in kidneys from patients with severe kidney fibrosis. (A) miR-21 expression obtained by real time PCR in kidneys from patients with severe kidney fibrosis (cases n = 11) versus normal human kidneys (controls n = 12). RNU6B was used as normalizer. Data are expressed as mean ± SEM. * p<0.05. (B) *In situ* hybridization assay was performed to determine to determine the stromal localization of miR-21 and its colocalization with α-SMA in the kidney from detransplanted patients. Results represent one out of three independent experiments.

Interestingly, miR-21 expression was strongly up-regulated in kidneys explanted from renal transplanted patients and exhibiting severe fibrosis on graft biopsies (**Figure 4A**). This tissue expression was specifically localized in myofibroblasts-rich areas, as determined by α-smooth muscle actin immunostaining (**Figure 4B**).

### Elevated circulating miR-21 levels are associated with kidney fibrosis

**Figure 5:**
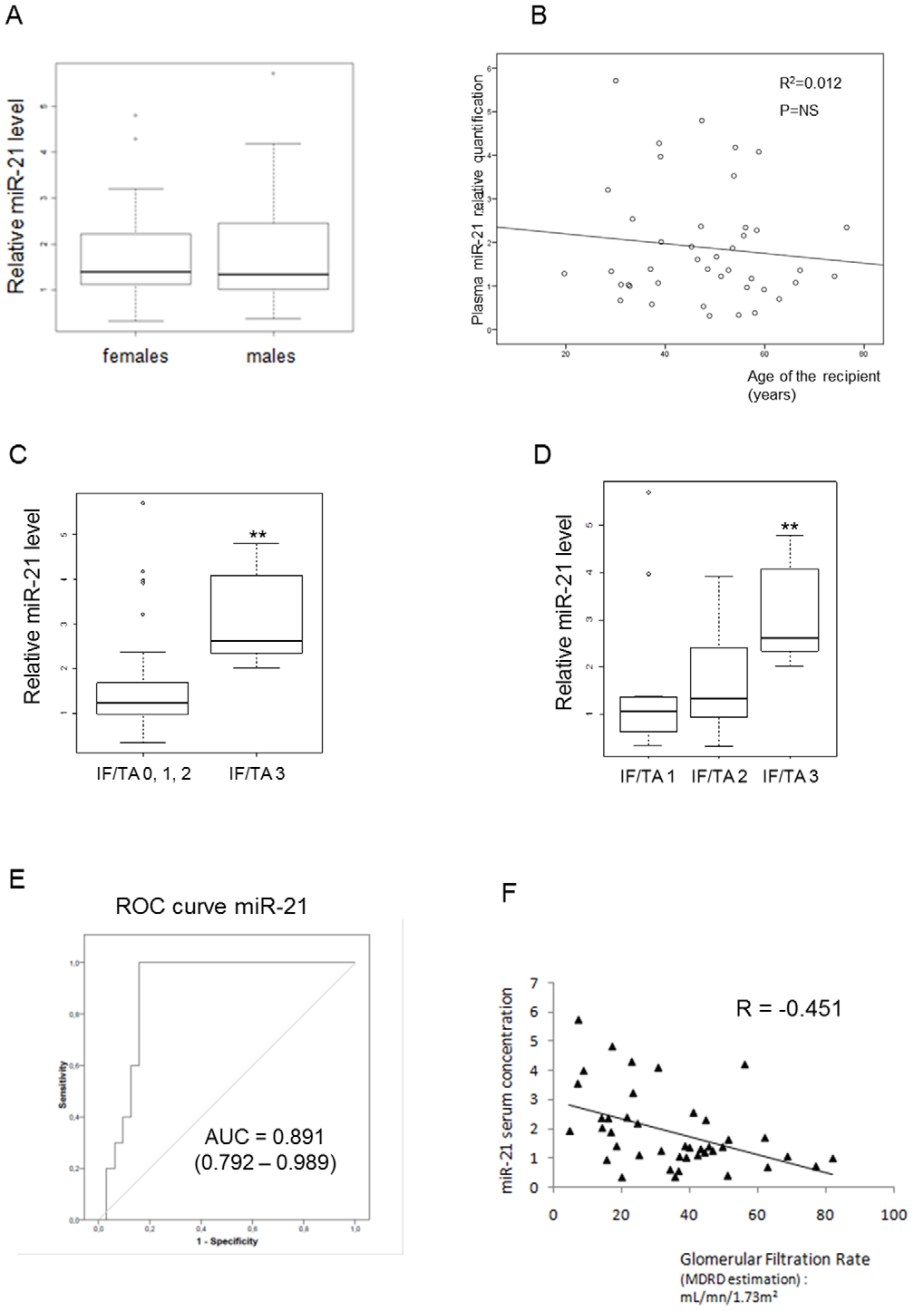
Circulating miR-21 is a potential biomarker of kidney fibrosis. (A, B) Circulating miR-21 levels are not affected by patient gender or age. (C, D) miR-21 circulating levels are increased in patients with high grade of Interstitial Fibrosis/Tubular Atrophy grade 3 (IF/TA grade 3). MiR-16 was used as normalizer. (E) Receiver operating characteristic curve analysis displaying the diagnostic power in predicting severe kidney fibrosis (IF/TA grade 3) of circulating miR-21 levels (area under the curve (AUC): 0.891). (F) Significant correlation (p<0.003, R = −0.451) between circulating miR-21 and glomerular filtration rate (based on MDRD formula) in patients with various grade of kidney fibrosis is shown. Data are expressed as mean ± SEM. ** p<0.01.

**Figure 6:**
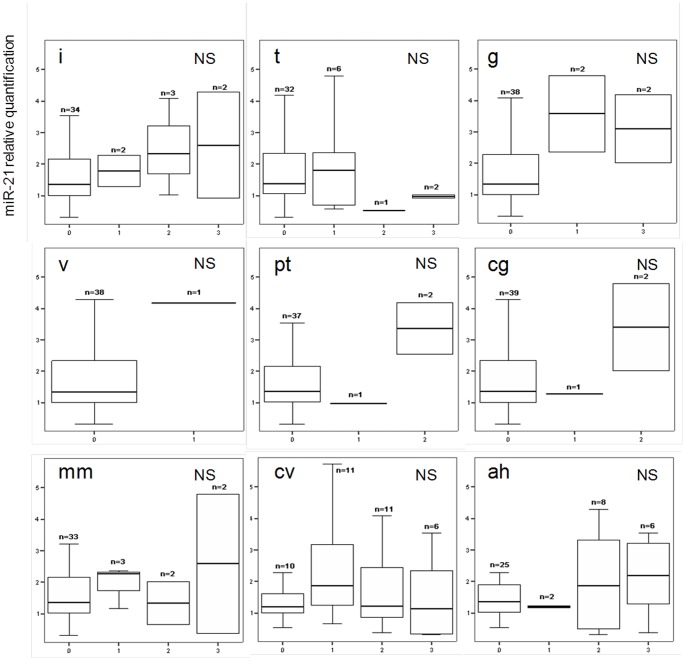
Circulating miR-21 levels are not associated with Banff criteria related to acute lesions or chronic vascular or glomerular lesions. i: Mononuclear Cell Interstitial Inflammation, t: Tubulitis, g: Glomerulitis, v: Intimal Arteritis, ptc: Peritubular Capillaritis, cg: Allograft Glomerulopathy, mm: Mesangial Matrix Increase, cv: Fibrous Intimal Thickening, ah: Arteriolar Hyaline Thickening. NS: not significant.

Patients were categorized in different groups according to renal allograft fibrosis score based on Banff classification as detailed in **Table 2**. To exclude potential biases, we determined to what extent serum miRNA levels were confounded with the baseline patient characteristics. Serum miR-21 levels were not different according to patient gender or age (**Figure 5A–B**). Serum levels of miR-21 in patients with severe fibrosis (IF/TA grade 3) were significantly elevated when compared to other patients (IF/TA grades 0, 1 and 2) (3.0±1.0 *vs* 1.5±1.2; p<0.001) (**Figure 5C**). As shown in **Figure 5D**, circulating miR-21 levels gradually increased with IF/TA fibrosis grade (R = 0.376, p = 0.01). The predictive power of circulating miR-21 levels for the presence of severe kidney fibrosis (IF/TA grade 3) was evaluated by ROC-analysis (**Figure 5E**). This revealed an area under the curve of 0.891 (95% confidence interval, 0.792–0.989). By contrast, circulating miR-21 levels were not correlated with other Banff criteria related to acute inflammation, chronic vascular or glomerular changes (**Figure 6**). Subsequently, we assessed the correlation between serum levels of miR-21 with patient renal function. MiR-21 correlated significantly to both estimated glomerular filtration rate (GFR) evaluated by aMDRD formula (regression coefficient R = −0.451, p = 0.003, **Figure 5F**) and plasmatic creatinine (R = 0.490, p<0.0001, not shown). In a model including IF/TA grade and estimated GFR, multivariate linear regression described independent associations between circulating miR-21 levels and IF/TA score (ß = 0.307, p = 0.03), as well as miR-21 levels and aMDRD (ß = −0.398, p = 0.006). By contrast, no correlation was observed between miR-21 levels and cellular lysis markers (as lactate dehydrogenase, alanine amino transferase and aspartate amino transferase) or C Reactive Protein.

**Table 2:** Patient demographics.

	IF/TA 0	IF/TA 1	IF/TA 2	IF/TA 3	p-value
n	13	12	7	10	ns
Male/Female (n)	9/4	8/4	2/5	4/6	ns
**Post-graft delay** (months)	19.0±6.2	35.9±37.2	66.2±43.4	70.9±56.3	p = 0.002
**Age** (recipient, years).	47.2±13.0	49.3±15.4	42.9±15.4	42.9±15.4	ns
eGFR (MDRD, mL/min/1.73m^2^)	34±19	40±21	42±19	24±11	ns

ns: not significant, eGFR: estimated Glomerular Filtration Rate, MDRD: Modification of Diet in Renal Diseases.

## Discussion

The discovery of serum miRNAs as potential biomarkers holds great promise to overcome the difficulties of collecting tissue samples by an invasive process. In particular, as circulating miRNAs are reliably stable and can be detected with high sensitivity and specificity [25]–[26], recent studies have reported that, in response to tissue injury, miRNAs expressed in specific cell types can be released into the bloodstream [20], reflecting therefore tissue damage.

In this study, we first investigated whether the UUO mouse model accurately reflected the changes in miRNA expression occurring during human kidney fibrosis. Overall, our data showed a good concordance between miRNAs differentially expressed in human and mice fibrotic kidneys, suggesting therefore that the UUO mouse model is clinically relevant to investigate miRNA pathogenic functions. Although microarrays were performed at late stage of fibrosis (28 days after UUO), our data are consistent with previous reports based on mice that underwent a shorter obstruction period [27]–[29]. In line with previous findings, miR-29 family members (i.e. miR-29b and miR-29c), which are involved in the regulation of ECM component synthesis, especially collagen [30]–[32], are under-expressed 28 days after UUO. We also observed a down-regulation of miR-192, a miRNA exhibiting both pro- and anti-fibrotic properties depending of cell phenotype [33]. Nevertheless, controversial results have been reported, as Zarjou *et al.* [27] also observed a down-regulation of this miRNA whereas Chung *et al*. [34] found an up-regulation of miR-192 using the same experimental mouse model.

Given the established role of miR-21 in tissue fibrosis [14], [23]–[24], [27]–[28], we decided to focus on this miRNA by assessing whether miR-21 in addition to its profibrotic effects was a reliable non invasive blood marker of kidney fibrosis.

MiR-21 is one of the most extensively studied miRNA. Previous reports have indeed shown its consistent up-regulation in various disease conditions including cancers [35]–[37]. In the context of fibrosis, recent reports demonstrated a major role of miR-21 in mediating the pathogenic activation of lung, kidney and cardiac fibroblasts and ultimately fibrosis [27], [38]–[40]. Importantly, miR-21 could also be a novel promising therapeutic target to treat fibrosis. Indeed, *in vivo* modulation of miR-21 using antisens oligonucleotides strategy may attenuate the manifestation of chronic fibrosis [28]–[29], [41]–[42].

In the present study, our data confirmed the central pathogenic role played by miR-21 to orchestrate the molecular events leading to kidney fibrosis. MiR-21 expression was consistently induced in both human and mouse fibrotic kidney, especially in myofibroblasts-rich areas. In particular, miR-21 expression was well correlated with disease progression in the experimental mouse model and was significantly up-regulated as early as day 4 following UUO. Therefore, altered expression of miR-21 is likely to represent an early molecular event driving the fibrosis process rather than a secondary effect of the long-standing disease process.

Although miR-21 functions have been extensively characterized in tissue fibrosis, these previous studies largely focused on miR-21 tissue expression and did not evaluate the potential of circulating miR-21 level as a biomarker of tissue fibrosis [28], [40]. Only Villar *et al*. (2012) described a correlation between myocardial and circulating miR-21 levels in aortic stenosis patients with myocardial fibrosis [43]. Therefore, we tested the hypothesis that circulating miR-21 levels are associated with renal fibrosis and discriminate patients by fibrosis grade. Patients included in this study were renal transplant recipients treated with Tacrolimus, an anti-calcineurin known for its pro-fibrotic effects [44]–[45]. To our best knowledge, we report for the first time that miR-21 circulating levels are not only significantly increased in patients with severe IF/TA grade but also gradually increase with IF/TA fibrosis grade. This correlation was specific of kidney fibrosis as no other significant association was found with other histological changes related to acute inflammation, chronic vascular or glomerular alterations. This strongly suggests that the increased expression of miR-21 observed in serum reflects the proportion of kidney tissue damaged by active fibrosis. Noteworthy, we observed a significant inverse correlation between circulating miR-21 levels and patient renal function status evaluated by aMDRD (abbreviated Modification of Diet in Renal Disease) formula. Nevertheless, in a multivariate analysis, circulating miR-21 levels remain associated to kidney fibrosis grade, independently of renal function.

In conclusion, our results defined miR-21 as a reliable serum marker of severe kidney fibrosis in renal transplanted patients. This study highlights that assessing circulating miRNAs is a promising non invasive approach that may help to stratify patients according to kidney fibrosis grade. From this point of view, large scale assessment of circulating miRNA levels using high throughput technologies will undoubtly provide new specific markers relevant for clinicians.

## Methods

### Ethics Statement

Patients provided their written informed consent. This study was performed in accordance with the Declaration of Helsinki and was approved by our local ethics committee (CPP Nord-Ouest IV).

All animal care and experimental protocols were approved by the Institutional Animal Care and Use Committee (IACUC) of Lille 2 University (Protocol Number: CEEA 162011). Manipulators carried out all experimental protocols under strict guidelines to ensure careful and consistent handling of the mice.

### Patients

42 renal transplanted recipients uniformly treated with Tacrolimus were included. During the follow-up, biopsies were performed routinely three months after transplantation (protocol biopsies) or later on clinical indication (for cause biopsies), as described in our local regular protocol of renal transplant care.

Renal fibrosis severity was evaluated according to histological criteria (interstitial fibrosis (ci) and tubular atrophy (ct) scores) defining Interstitial Fibrosis/Tubular Atrophy (IF/TA, 2007 Banff classification) [46]. Patients were selected according to IF/TA score in order to get homogeneous groups. Sera were collected at the time of biopsies.

Non-pathological renal parenchyma samples were collected from twelve nephrectomies performed for renal or urinary tract cancer in patients with normal renal function. Mirror samples were analyzed by a pathologist to ensure the absence of lesion. Fibrotic renal parenchyma samples were obtained from eleven explanted renal allografts.

### Mouse model of kidney fibrosis

All animal care and experimental protocols were conducted according to European, national and institutional regulations (Protocol Number: CEEA 162011). Manipulators carried out all experimental protocols under strict guidelines to ensure careful and consistent handling of the mice. 9–12 weeks old male C57BL/6 mice were purchased from Charles River. Mice underwent anesthesia by intraperitoneal injection of pentobarbital (50 mg/kg body weight) and were subjected to Unilateral Ureteral Obstruction (UUO). After standard laparotomy, the left proximal ureter was exposed and ligated with 4–0 silk at two points. The sham operation consisted of a similar identification of the left ureter without ligation.

### Histopathology

Human and mouse kidney samples from non-pathological and fibrotic kidney specimens were formalin-fixed and paraffin-embedded. Three-micrometer-thick sections were stained with hematoxylin and eosin as well as Masson's trichrome to assess the degree of fibrosis. Grading was performed according to updated Banff 07 classification [46].

### Mouse renal fibroblast primary culture

Mouse kidneys were dissected in 1 mm3 fragments. Fragments were incubated 30 min at 37°C in 5 mL of complete Dulbecco's Modified Eagle Medium (DMEM) containing 10% fetal bovine serum (FBS), 1% penicillin/streptomycin and 2 mg/mL collagenase IV (200 U/mL, Invitrogen). This step was performed three times and the suspension was filtered after each digestion step on 70 µm filters. Cells were centrifuged 5 min at 1200 rpm and washed twice with Phosphate Buffer Saline (PBS). Cells were cultured with complete DMEM (10% FBS) at 37°C with 5% v/v CO2 in a humidified atmosphere. One hour after plating, medium was changed to remove non adherent epithelial cells.

### Pre-miRNAs overexpression in kidney fibroblasts

Pre-miR-21 and control miRNA (miR-Neg # 1) were purchased from Ambion. Primary mouse renal fibroblasts were grown in 10% FBS in DMEM and transfected at 50% confluency in 6-well plates using Lipofectamin RNAi MAX™ (Invitrogen) with pre-miRNA at a 10 nM final concentration. Recombinant TGFβ was purchased from Sigma-Aldrich.

### miRNAs isolation from serum

Total RNAs, including miRNAs, were extracted from 200 µL of serum using miRNeasy Mini kit (Qiagen) with few modifications. In particular, 4 µg MSII RNA (Roche) were added to samples chloroform extraction.

### Tissues and cells RNA extraction

Total RNA were extracted from tissue and cell samples with the miRNeasy Mini kit (Qiagen). Integrity of RNA was assessed by using an Experion Automated Electrophoresis System (Biorad) (RQI above 8).

For human formalin-fixed and paraffin-embedded kidney samples, total RNA was obtained using the RecoverAll™ Total Nucleic Acid Isolation Kit (Ambion).

### Array analysis

MicroRNA profiling was performed with an Agilent GeneChip® Mouse miRNA Microarray containing 567 microRNAs (Sanger miRbase release 10.1), in accordance with the protocol described by the manufacturer (Agilent). MicroRNA microarray data were log2 transformed and normalized to the mean of each array, and a t-test was used to identify those microRNAs that were differentially expressed (p<0.01) between injured kidneys and controls. The experimental data and microarray design have been deposited in the NCBI Gene Expression Omnibus (GEO) (http://www.ncbi.nlm.nih.gov/geo/) under serie GSE39831.

### Quantitative RT-PCR

#### Mature miRNAs expression

MiR-21 expression was evaluated using TaqMan MicroRNA Assay (Applied Biosystems) as described in their protocol. Real-time PCR was performed using Master Mix II, no UNG (Applied Biosystems) and Step One Plus Real Time PCR System. Expression levels of mature microRNAs were calculated based on the comparative threshold cycle method (2^−ΔΔCT^) [47].

#### Gene expression

Expression levels of fibronectin, collagen 1, TGFβ and αSMA (ACTA2) were analyzed using TaqMan MicroRNA Assay (Applied Biosystems) according to the manufacturer's instructions. Real-time PCR was performed using TaqMan® Gene Expression Master Mix (Applied Biosystems) and Step One Plus Real Time PCR System. Expression levels of mRNA were assessed using the comparative threshold cycle method (2^−ΔΔCT^) [47]. PPIA (cyclophilin A) was used as normalizer.

### Immunohistochemistry

Immunohistochemical study was performed on an automated immunostainer (Benchmark XT, Ventana) with the XT ultraview diaminobenzidine kit. Dewaxed samples were subjected to antigen retrieval in Tris-EDTA buffer pH 9 for 30 min, then incubated with the primary antibody anti-human alpha smooth muscle antigen (clone 1A4, DAKO; dilution 1/200). Negative controls were performed by omitting primary antibodies.

### *In situ* hybridization

*In situ* hybridization of miR-21 was performed using double DIG-labeled LNA probes (Exiqon, Woburn, MA). Paraffin-embedded mouse kidneys were dewaxed in xylene and rehydrated in descending grades of alcohol. Slides were then washed in PBS (pH 7.5) and permeabilized by incubating in proteinase K (Exiqon) for 15 min at 37°C. The slides were again washed in PBS, and prehybridized in hybridization buffer (Exiqon) in a humidified chamber. The 5′ DIG-labeled LNA probes were then added to the sections at a 80 nM concentration and incubated 2 hours at 50°C in a humidified chamber. Slides were rinsed in 5X SSC, 1X SSC and 0.2X SSC solutions at the same hybridization temperature and rinsed again with 0.2X SSC at room temperature. This was followed by blocking with 2% sheep serum, 2 mg/mL Bovine Serum Albumine in PBS +0.1% Tween 20 (PBST) and incubation with anti-DIG-AP Fab fragments antibody (1∶800) (Roche Applied Sciences) for 2 hours at room temperature. After washing in PBST, the color reaction was carried out by incubation in 5-bromo-4-chloro-3-indolyl phosphate (BCIP)/nitro blue tetrazolium (NBT) color solution (Roche Applied Sciences) with 1 mM levamisole overnight at room temperature. The color reaction was stopped after observation of sufficient development of blue precipitate by washing with PBST. Slides were then counterstained with Fast-Red.

### Statistical analysis

Results of experimental data are given as mean ± Standard Error of the Mean (S.E.M). Statistical significance between experimental groups was assessed using ANOVA, unpaired Student's t-test or non parametric test. Correlations between serum levels of miRNA and clinical values were determined by calculating the Pearson correlation coefficient. In order to appreciate the accuracy of miR-21 as kidney fibrosis marker (IF/TA grade 3), a Receiver Operating Characteristic (ROC) curve was generated. To analyze association between miR-21 and covariables, we performed multivariable linear regression.

Statistical analyses were performed using the SPSS package, version 15.0 for Windows (Chicago, Illinois, USA). Significance was assumed at a p level of less than 5%.
